# Challenges of Conducting Risk‐Benefit Analysis of Early Phase Clinical Trials: Results of a National Survey of IRB Chairs

**DOI:** 10.1002/eahr.60024

**Published:** 2025-11-12

**Authors:** Christine M. Baugh, Dragana Bolcic‐Jankovic, Mark Fedyk, Mark Yarborough, Spencer Phillips Hey, Insoo Hyun, Jonathan Kimmelman, Eric G. Campbell

**Affiliations:** ^1^ Assistant professor at the University of Colorado Anschutz School of Medicine in the Department of Medicine in the Division of General Internal Medicine and Center for Bioethics and Humanities at the University of Colorado; ^2^ Senior research fellow at the Center for Survey Research at the University of Massachusetts Boston; ^3^ Professor in the Division of General Medicine and Bioethics in the Department of Internal Medicine at the School of Medicine & Betty Irene Moore School of Nursing at the University of California, Davis; ^4^ Professor emeritus in the division of general medicine and bioethics in the Department of Internal Medicine at the School of Medicine at the University of California, Davis; ^5^ Chief science officer at Prism Analytic Technologies; ^6^ Director of the Center for Life Sciences and Public Learning in the Museum of Science, Boston and an affiliate of Harvard Medical School's Center for Bioethics; ^7^ Professor in the Department of Equity, Ethics and Policy at the School of Population and Global Health at McGill University; ^8^ Professor at the University of Colorado Anschutz School of Medicine in the Department of Medicine in the Division of General Internal Medicine and Center for Bioethics and Humanities at the University of Colorado

**Keywords:** human research ethics, institutional review boards (IRBs), clinical trials, early phase clinical trials, risk‐benefit analysis

## Abstract

Institutional review boards (IRBs) are charged with conducting risk‐benefit analysis for early phase clinical trials that often involve high levels of uncertainty regarding a trial's potential risks and benefits. Our study used a survey of IRB chairs to explore how IRBs conduct risk‐benefit analysis, the unique facets of risk‐benefit analysis for early phase clinical trials and specifically for early phase neurology trials, and what facilitates high‐quality risk‐benefit analysis. The survey measured IRB chairs’ perceived difficulty, preparedness, processes, and satisfaction with risk‐benefit analysis for early phase trials. The survey was completed by 148 of the 259 eligible IRB chairs for a response rate of 64.6%. Two‐thirds of respondents found risk‐benefit analysis for early phase clinical trials more challenging than for later phase trials. Ninety‐one percent of respondents felt that their IRB did an “excellent” or “very good” job conducting risk‐benefit analysis, but more than one‐third of respondents did not feel “very prepared” to conduct key aspects of risk‐benefit analysis. Over two‐thirds of respondents reported that additional resources, like a standardized process for conducting risk‐benefit analysis, would be mostly or very valuable. Our results suggest that conducting risk‐benefit analysis for early phase clinical trials generally and early phase neurology trials specifically is challenging for IRBs. One‐third of respondents reported that they lack preparation for assessing the scientific value of these types of trials and the risks and benefits to research participants, and a majority desire additional support.

Institutional review boards (IRBs) serve a key function of ensuring there is an appropriate balance of risks and benefits in the research studies they review, including clinical trials of new drugs and biologics. Risk‐benefit analysis is a core duty of ethics review, made explicit in the Common Rule, the *Belmont Report*, and the Declaration of Helsinki.^1^ Tasks associated with risk‐benefit analysis include, but are not limited to, identifying all risks associated with trial participation, estimating their probabilities and severity, judging the adequacy of the measures in place to prevent or minimize harm(s), and considering risk against the prospect of direct and social benefit.^2^ According to the *Belmont Report*, risk‐benefit analysis should strive to be accurate, support transparent and nonarbitrary ethical decisions, and that “the nature, probability and magnitude of risk should be distinguished with as much clarity as possible.”^3^ Risk‐benefit analysis supports minimization and balancing of the risks of participating in research against direct benefit to research participants and the scientific value of the research. Careful weighing of the quality and strength of evidence is essential to an ethically responsible risk‐benefit analysis and the evidence about and consideration of potential benefits should be on equal footing with the evidence about and consideration of potential risks.

U.S. agencies that govern research with humans have provided little substantive guidance to IRBs about how to conduct risk‐benefit analysis. IRBs thus have complete discretion in terms of how they perform risk‐benefit analysis, including what they consider and how they balance research risks against the potential research benefits to study participants.^4^ Some may view this discretion as necessary to allow for the vagaries of different clinical trial designs and the characteristics of the disease under study. However, others worry that discretion compromises the integrity and effectiveness of the risk‐benefit process and thus puts research participants at risk for participation in clinical trials that fail to adhere to ethical standards.^5^ Despite the critical role IRBs play in ensuring the ethical conduct of clinical trials, little is known about the quality and function of IRBs in the United States.^6^ In February 2023, the U.S. Government Accountability Office (GAO) published a report that called for action from both the Department of Health and Human Services (DHHS) and the U.S. Food and Drug Administration (FDA) to enhance their oversight and review of IRBs.^7^

The impact of the relative lack of substantive guidance for conducting risk‐benefit analysis, as well as the lack of oversight and insight into IRB practices, is perhaps most vexing for early phase clinical trials (first‐in‐human, phase 1 and phase 2 trials). In numerous cases, early phase trials have been launched on the backs of equivocal, or even unfavorable, supporting preclinical evidence.^8^ This is especially true in neurology, given the high rates of drug attrition during drug development for neurological disorders.^9^ In all early phase clinical trials, IRBs must rely heavily or exclusively on preclinical research, requiring them to extrapolate risks and potential benefits to a human population.^10^ This challenge is amplified in the case of neuroscience preclinical studies, as the unique nature of human cognition, behavior, emotion, and volition means that there are no reliable animal models in which to study these phenomena.^11^ Additionally, preclinical studies are frequently subject to numerous problems with both design and reporting.^12^ Preclinical neuroscience studies are also plagued by publication bias in favor of positive studies.^13^ Further, many preclinical studies are designed to be hypothesis generating, rather than hypothesis confirming.^14^ To this end, the findings they report, though potentially promising, often cannot be definitively determined to be true or false, making failure likely in early phase trials

To our knowledge, how IRBs approach risk‐benefit analysis for early phase clinical trials—in general or in neurology specifically—has not been assessed in a national sample of IRBs.^15^ We conducted a survey of IRB chairs in U.S. medical schools and health systems to empirically explore IRBs’ approaches to reviewing early phase clinical trials.

**IRB chairs feel additional measures would be helpful in conducting risk‐benefit analysis for early phase clinical trials.**

## STUDY METHODS

We used a multistep process to create our sample that followed the sampling process of earlier studies developed by Campbell and colleagues.^16^ First, using the National Institutes of Health (NIH) RePORTER, we pulled the 2022 NIH fiscal year institutional ranking for all NIH‐funded domestic institutions of higher education (DHEs, n = 526) and all independent hospitals (n = 79). Second, we selected the top 100 NIH‐funded DHEs that also have a medical school and the top 15 NIH‐funded independent hospitals from this list. We then excluded laboratories (n = 23), psychiatric‐specific (n = 1), ear care center‐specific (n = 1), and cancer‐specific (n = 4) centers from the list of independent hospitals, and we excluded DHEs that did not have a medical school or had their respective medical school listed independently, also affiliated with a DHE ranked higher on the list (n = 7). Third, we used the Institutional Organization Number of each selected DHE and independent hospital to identify every active IRB within each institution from the publicly available online database on the webpage of the Office of Human Research Protections (OHRP). This generated 384 DHE IRB numbers and 40 independent hospital IRB numbers, for a total of 424 unique IRBs. Fourth, we requested the names of the IRB chairs from each of the 424 IRBs from the OHRP; the OHRP provided IRB chair information for 420 IRBs (380 DHE IRBs, and 40 independent hospital IRBs). Lastly, we cleaned the data first by identifying and removing duplicate names and then by looking up each IRB chair's name at their respective institution to find more complete contact information. This process yielded a final clean sample of 326 IRB chairs, including 296 from DHEs and 30 from independent hospitals.

Survey development followed a standard multistep process.^17^ First, we constructed a list of data elements central to the research questions. Next, we identified existing measures that assessed these elements. When no appropriate measures existed, we created survey items de novo and iteratively refined them as a team. When the survey was nearly completed, we conducted cognitive interviews with seven eligible volunteers to identify survey items that were unclear, difficult to answer, or were not interpreted as anticipated by members of the target population. The survey was revised based on the cognitive interviews. Finally, we tested the survey under field conditions by sending a first wave of surveys, including $40 participation incentives, by U.S. Postal Service (USPS) priority mail to a pre‐test sample of 40 individuals. The mail survey invitation included a weblink to complete the survey online. The actual survey is included in the appendix (available online; see information about accessing this material in the “Supporting Information” section at the end of this article). This study was ruled exempt by the UC Davis IRB (#1834845‐4) and the UMass Boston IRB (#2021136).

The survey was administered by the Center for Survey Research at the University of Massachusetts Boston between November 2022 and April 2023. An 8‐page questionnaire was mailed using USPS priority mail, along with a cover letter (with a link to an online version of the survey), a factsheet, a postage‐paid return envelope, and a $40 incentive. The follow‐up efforts to nonrespondents included a second mailing, reminder calls, and a final mailing. The second and final mailings were mailed using first‐class mail and did not include cash incentives.

Given that the survey was identical in both the pre‐test and the main data collection, and the fact that 326 was the universe of those who met the study criteria, the survey data from both survey phases were combined for this analysis. Sampled members were deemed ineligible for the study if they self‐reported not being a chair, a co‐chair, or a vice‐chair of an IRB in the past two years, not having experience reviewing early‐phase clinical trials, if they were no longer at the sampled institution, if they were retired, deceased, away for the duration of the study, or could not be located by mail, reminder calls, or internet searches. The total eligible sample was 259 cases. A total of 148 IRB chairs, co‐chairs, or vice‐chairs completed the survey (61% on paper and 39% online), resulting in an overall 64.6% response rate (AAPOR#3 [the American Association of Public Opinion Research #3 response rate calculation includes an estimation of eligible unknown cases]).

We compared the survey respondents to our sample on three key characteristics: the type of institution (medical school versus hospital), NIH ranking tier in tertials based on NIH awards in 2022, and geographic region (see table 1). This analysis shows our respondents very closely resemble the eligible sample with the largest difference being a 3.9 percentage point difference among respondents in the highest NIH ranking tier.

**Table 1:** Characteristics of Respondents Compared to the Sample

	*N (%) respondents*	*N (%) of eligible sample*	*Percentage point difference*
**IRB setting**			
Medical school	131 (88.5%)	233 (90.0%)	‐1.5
Hospital	17 (11.5%)	26 (10.0%)	1.5
**NIH ranking tertile** *			
Top	68 (45.9%)	129 (49.8%)	‐3.9
Middle	41 (27.7%)	(69) (26.6%)	1.1
Bottom	39 (26.4%)	(61) (23.6%)	2.8
**Geographic region**			
Northeast	40 (27.0%)	73 (28.2%)	‐1.2
Midwest	24 (16.2%)	44 (17.0%)	‐0.8
South	54 (36.5%)	87 (33.6%)	2.9
West	30 (20.3%)	55 (21.2%)	‐0.9

* Based on total awards from the NIH to the institution in 2022. This only includes medical schools and not hospitals.

To preserve the anonymity of respondents, several data protection steps were implemented. First, the NIH ranking was only provided for the sample members from medical schools but not from hospitals given the small size of the hospital stratum. Second, the detailed answers on the type of IRB setting were only provided as frequency distributions and not included in the dataset; instead, the answers in the dataset were dichotomized into medical school versus not medical school. Third, the race variable was also not included in the dataset and was only provided as a frequency distribution given the small sample size of some racial categories.

### Key definitions

The survey stated, “Early Phase Trials are usually Phase 1 but sometimes Phase 2 clinical trials of drugs or biologics being studied for the first time in patients with a particular condition.” In addition, we defined risk‐benefit analysis as “the process used by your IRB to determine whether or not the ratio of risks and benefits for a given clinical trial is reasonable. By reasonable, we mean that in federal regulations IRBs are required to determine that the research has a ‘reasonable ratio’ between the risks and benefits of conducting a study.”

### Difficulty performing risk‐benefit analysis

On the survey we asked, “Compared to later phase trials, how difficult is it for your IRB to conduct a risk‐benefit analysis of early phase clinical trials?” The response categories were “a lot more difficult,” “a little more difficult,” “about the same,” “a little easier,” and “a lot easier.” Because no respondents answered “a little easier” or “a lot easier,” the resulting variable had three categories corresponding to “a lot more difficult,” “a little more difficult,” and “about the same.”

### Preparedness to conduct risk‐benefit analysis

On the survey we asked, “Overall, how prepared is your IRB to do each of the following for protocols for early phase clinical trials? By prepared we mean your IRB has sufficient expertise and information to … analyze the potential direct benefits for participants; analyze the potential for knowledge benefits of the trial; analyze the risks; determine if the risks have been adequately minimized; determine if the risks are reasonable in relation to the benefits.” The response categories were “very prepared,” “mostly prepared,” “a little prepared,” and “not at all prepared.” We then coded “not at all prepared” responses as 0, “a little prepared” coded as 1, “mostly prepared” coded as 2, and “very prepared” coded as 3. Because completing a risk‐benefit analysis requires all the sub‐components in the preparedness scale, we then created a separate variable in which we summed the scores for each of the five questions yielding an overall composite preparedness score ranging from 0‐15.

### Risk‐benefit analysis process

On the survey we asked, “When conducting a risk‐benefit analysis of early phase clinical trials to what extent does your IRB rely on … Information from relevant pre‐clinical studies that is included in the investigator's brochure provided by the sponsor; information from preclinical studies that supplements the information in the investigator's brochure provided by the sponsor; expertise of IRB members; and ad hoc expertise from outside of your IRB.” The response categories were “to a great extent,” “to some extent,” “very little,” and “not at all.” We then used a top box coding approach to create four separate variables for each question, with “to a great extent” coded as 1 and all other responses coded as 0.

### Quality of risk‐benefit analysis

On the survey, we asked, “Overall how good of a job does your IRB do when conducting a risk‐benefit analysis of early phase clinical trials?” The response categories were “excellent,” “very good,” “good,” “fair,” and “poor.” We then used a top box coding approach with “excellent,” “very good” responses coded as 1, and all other responses coded as 0.

The survey data were analyzed using standard biostatistical tests and conducted in R Studio. Univariate description statistics including n's and percentages were computed for categorical variables. Means and standard deviations were computed for continuous variables. Standard chi‐square testing was used for bivariate analyses of categorical variables. Given the distribution of the composite variable measuring preparedness to perform risk‐benefit analysis, we used the Kruskal‐Wallis (nonparametric) chi‐square ranked sum test. All differences at the p < .05 level were considered significant.

## Study Results

Table 2 shows the characteristics of the respondents. Approximately two‐thirds of respondents were male (65%). Due to the extremely small number of non‐white and Hispanic respondents, we are unable to report that data as doing so would potentially identify certain respondents. Of the respondents, 41% reported they had been a principal investigator and/or a co‐investigator for at least one early phase clinical trial.

**Table 2:** Respondent Characteristics

	*N (%)*
**IRB position**	
Chair	125 (87%)
Co‐chair or vice‐chair	19 (13%)
**Gender**	
A woman	51 (35%)
A man	95 (65%)
**Principal iInvestigator or co‐investigator experience for early phase clinical trial**	
Yes	61 (41%)
No	87 (59%)
**IRB setting**	
Medical school	125 (86%)
Not medical school	20 (14%)
**New protocols reviewed per year**	
Less than 100	75 (52%)
100‐199	41 (28%)
200‐499	19 (13%)
500 or more	9 (6%)
**Experience reviewing early phase clinical trials**	
Yes	143 (100%)
**New early phase clinical trials protocols reviewed per year**	
None	9 (6%)
1‐4	34 (23%)
5‐9	32 (22%)
10 or more	71 (49%)
**IRB reviews neurological clinical trials**	
Yes	130 (89%)
No	16 (11%)
**IRB members with expertise in neurological diseases**	
Yes	103 (79%)
No	27 (21%)
**Early phase neurological clinical trials reviewed per year**	
None	26 (20%)
1‐4	63 (48%)
5‐9	29 (22%)
10 or more	12 (9%)

More than two thirds of respondents (68%) chaired medical school‐based IRBs with the remainder (32%) chairing IRBs in independent hospitals. The IRBs varied in terms of the volume of new early phase clinical trial protocols reviewed per year with 6% reviewing 0 early phase protocols in the last year, 23% reviewing 1‐4 protocols, 22% reviewing 5‐9 protocols and 49% reviewing 10 or more (see table 1). The IRBs of respondents also varied in the number of new early phase trials in neurology, with 20% reviewing no such protocols, 48% reviewing 1‐4 protocols, 22% reviewing 5‐9 protocols and 9% reviewing 10 or more protocols. Of the respondents, 79% reported having IRB members with expertise in neurologic diseases.

Two thirds of respondents (66%) reported that it was more difficult to conduct a risk‐benefit analysis for early phase clinical trials than for later phase trials. Almost all respondents (91%) felt their IRB did an “excellent” or “very good job” conducting the risk‐benefit analysis for early phase trials. On the survey, we asked about the sources of information that IRBs relied on when conducting a risk‐benefit analysis. IRBs most frequently relied on the study sponsor's Investigator's Brochure (72.6%), followed by the individual expertise of IRB members (63.4%), supplemental information provided by investigators (44.8%), and ad hoc expertise from individuals outside the IRB (8.2%). Nearly all respondents (91%) felt that a trial sponsor having an investigational new drug application (IND) approved by the FDA provided some assurance that a study had a favorable balance of risks and benefits. Further, 86.5% of IRBs prioritized protecting patients from the risks posed by the research over ensuring patients have access to investigational drugs/biologics or advancing the development of new drugs/biologics (8.1%).

On the individual measures of preparedness, between a third to half of respondents said they were not “very prepared” to conduct key aspects of risk‐benefit analysis. For example, 42% did not state they were “very prepared” to analyze the potential knowledge benefits of the trial. Slightly less did not feel they were “very prepared” to analyze the potential direct benefits (38%) or risks (38%) to study participants. Overall, one third of respondents did not state they were “very prepared” to determine if the risks have been adequately minimized and to determine that the risks are reasonable in relation to the benefits.

Eighty‐nine percent of respondents reported that their IRB reviewed protocols for neurological studies. Of these, when reviewing protocols for neurological diseases, 72% of respondents rarely sought expertise outside of their IRB. In the process of conducting a risk‐benefit analysis for early phase neurological clinical trials, 90% of IRB chairs stated they discuss the preclinical studies that support the clinical trial to be considered for approval. Seventy‐eight percent of respondents said their IRB used a standard review template and no additional standardized processes were used when conducting a risk‐benefit analysis for early phase neurological clinical trials.

**Figure 1:**
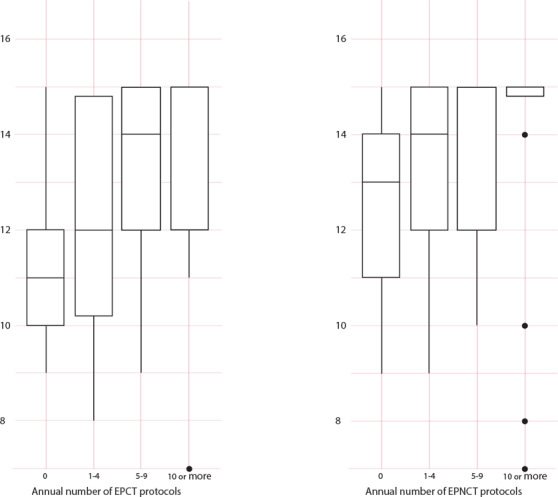
Relationship between EPCT and EPNCT Protocol Review Volume and IRB Preparedness* * Boxplot of volume of relationship between the number of early phase clinical trial (EPCT) protocols reviewed and IRB preparedness (both EPCT and early phase neurological clinical trials [EPNCT] together in a composite figure).

Using the composite preparedness variable, the mean preparedness score was 13.1 with a standard deviation of 2.0. Lower composite preparedness was significantly associated with the low volumes of review of protocols for early phase clinical trials (in general) reviewed in the previous year. For example, the mean preparedness score for IRBs that had not reviewed any early phase protocols in the last year was 11.4 compared to 12.1 for those that reviewed 1‐4 protocols, 13.1 for those that reviewed 5‐9, and 13.6 for those that reviewed 10 or more protocols (p < .001 from Kruskal‐Wallis ranked sum chi‐square; see figure 1).

The volume of protocols for early phase clinical trials in neurology was also related to preparedness to conduct risk‐benefit analysis for these studies (see figure 1). For IRBs that reviewed no protocols for early phase trials in neurology in the last year, the average preparedness score was 12.4, compared to 13.1 for those that reviewed 1‐4, 13.6 for those that reviewed 5‐9, and 13.9 for those that reviewed 10 or more (p < .001 from Kruskal‐Wallis ranked sum chi‐square). No other IRB or respondent characteristics were found to be significantly related to overall preparedness.

**Figure 2:**
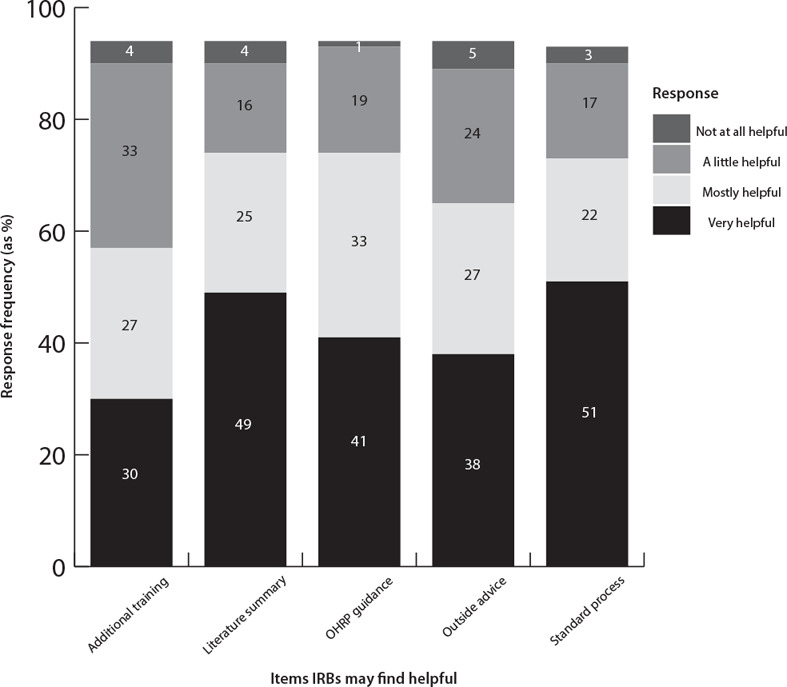
How Helpful IRBs Would Find Additional Supports When Reviewing EPNCTs* * Bar chart for all response options for question re: what IRB would find helpful? with each bar broken up (separate colors) by Likert response categories; all bars total 100%.

More than two‐thirds of IRB chairs reported that it would be mostly or very helpful to have additional resources when conducting risk‐benefit analysis for early phase trials in neurology (see figure 2). For example, 77% felt that having a standardized process for conducting risk‐benefit analysis would be mostly or very helpful. The same percentage felt similarly regarding having a computer program that summarizes the published literature on the benefits and potential risks of the drugs or biologics being studied as well as specific guidance from the OHRP (77%). In addition, 67% felt having an outside group such as a scientific review committee conduct a risk‐benefit analysis and advise the IRB would be helpful. Slightly more than half (57%) felt having additional training for IRB members regarding the review of early phase neurological clinical trials would be helpful.

## DISCUSSION

This study provides the first national data regarding how IRBs at medical schools and teaching hospitals conduct risk‐benefit analysis for early phase clinical trials. While it is reassuring that 92% of respondents felt their IRBs did a “very good” or “excellent” job conducting risk‐benefit analysis for early phase clinical trials, limited enthusiasm for this finding is potentially warranted given that one‐third of all respondents did not rate their IRB as “very prepared” to review early phase clinical trials, despite careful review of the trials being a prerequisite for the trials to be conducted. Further, it is somewhat hard to understand how the third of IRB chairs who report that their IRB is not very prepared to conduct risk‐benefit analysis for an early phase clinical trial can simultaneously feel their IRB does a good job conducting risk‐benefit analysis for early phase clinical trials. This may be due to a combination of social desirability bias, cognitive dissonance, and a potential over‐reliance on other entities involved in the research development process, such as the FDA and data safety monitoring boards. Future research should explore this potential inconsistency in respondents’ views. Although our findings are novel, we are not the first to identify areas of inconsistency or needed improvement in the quality of the IRB review process. Prior work has identified inconsistent application of Common Rule tenets,^18^ wide variation in approach and decisions,^19^ uncertainties around pediatric participation in research,^20^ lack of specific criteria used in decision‐making,^21^ IRB members who lack training on regulations^22^ or having insufficient expertise^23^ for review, and broad variation in the timeline of review,^24^ and fragmented research on the quality of research ethics review.^25^

In particular, some may find it concerning that one‐third of IRB chairs do not feel their IRB is “very prepared” to conduct risk‐benefit analysis for early phase trials. In our opinion, it is reasonable to expect that all IRBs should be “very prepared” to conduct risk‐benefit analysis for early phase clinical trials, as it is a key component of their regulatory charge and a sensible expectation by study participants. It is possible that this is an overestimate of preparedness (e.g., due to social desirability bias affecting responses), but it is conversely also possible that IRB chairs may not want to overstate their preparedness given the complexity of their work and the range of issues that come up in conducting a risk‐benefit analysis for these early‐stage protocols. That said, we find that IRBs that review low volumes of applications for early phase trials also report the lowest levels of preparedness to conduct risk‐benefit analysis for an early phase clinical trial. Given the specific challenges to understanding the risks of first‐in‐human studies, this relationship makes sense; IRBs that conduct risk‐benefit analysis for early phase trials infrequently are less prepared for these challenges. It is likely that there are other factors beyond the volume of such protocols that are reviewed that may explain this lack of preparedness, including but not limited to a lack of training and/or expertise in early phase clinical trials. Although there is certainly room for improvement, it is important to note that although one‐third of IRB chairs do not feel their IRBs are “very prepared” to conduct analysis, none indicated that their IRBs were unprepared and very few indicated that their IRBs were only a little prepared. Future research should explore the causes and potential implications of this lack of preparedness on IRB oversight of early phase clinical trials.

The data presented above constitute the first empirical confirmation in the United States of IRB chairs’ belief that conducting risk‐benefit analysis for early phase clinical trials is more difficult for IRBs than conducting risk‐benefit analysis for later phase trials. This difficulty may be due to informational challenges/deficits. For example, often in early phase studies, there are limited data from preclinical trials to adequately understand the potential benefits and risks of a proposed trial. This informational challenge is supported by our finding that only half of the respondents felt that the preclinical studies provided sufficient information to understand the risks or the potential benefits of the early phase trial being reviewed. Prior work has suggested that informing participants of research risks is widely considered very important by IRB members;^26^ without sufficient knowledge of these risks, meaningfully communicating them to participants is difficult or impossible.

This research sheds light on the potentially problematic role that INDs play in IRBs’ risk‐benefit analysis. In this study, 91% of respondents reported that a protocol that has an IND provided some assurance that the risks and benefits of the proposed trial are adequately balanced. This reliance is inappropriate in that IRB chairs appear to assume that the FDA has weighed the risks and potential benefits and found them proportionate when considering the IND. However, as one of us has previously described, while the FDA is tasked with assessing risk, its mandate does not extend to weighing risks against potential benefits, the most prevalent of which is knowledge value.^27^ The FDA rarely, if ever, refuses to permit an early phase drug trial taking place because of concerns about the strength of supporting evidence about the study's risks and potential benefits.^28^ Moreover, drug regulators like the FDA lack the authority—and likely the resources and expertise—to balance scientific utilities of a trial against risks. Instead, this responsibility is delegated to IRBs.^29^

The findings of our study suggest that IRBs would like additional tools and help in conducting risk‐benefit analysis of early phase clinical trials. This is evidenced by the fact that more than two‐thirds to three‐fourths of IRB chairs responded that a number of interventions would be helpful in conducting risk‐benefit analysis for early phase clinical trials in neurology, including having a computer program that summarizes the published literature on the benefits and potential risks of the drugs or biologics being studied (77%); having specific guidance from the OHRP (77%); and having an outside group such as a scientific review committee conduct a risk‐benefit analysis and advise the IRB (67%). This is in line with prior research noting inconsistencies in standards applied in protocol reviews across IRBs.^30^

It is important to stress the difficulty of designing tools that address this need. The problem of describing the state of scientific knowledge in any one area of study—formally, the problem of aggregating scientific results—is not yet solved. Meta‐analyses are probably the most mature attempt at a solution. But requiring that all preclinical studies be amendable to future meta‐analyses would involve standardizing scientific methods in ways that would dramatically slow scientific progress. This is an important point because it means that, absent such a solution, the decision‐making of IRB members will be undetermined by available scientific evidence, leading to the prediction that other factors “fill in the gaps” when determining whether any given study should be approved. The presence of such “other factors” may account for some of the heterogeneity of the results discussed above—for example, why nearly all IRB chairs report that they are doing a “very good job” while about a third feel that they are not prepared to conduct risk‐benefit analysis. These results therefore raise the intriguing possibility that IRBs have developed internal (i.e., non‐public) criteria and processes that address the problem of underdetermination.

Despite the difficulties faced in designing the kinds of tools that IRB chairs report they would find useful, the fact remains that they have a nondelegable responsibility to conduct rigorous risk‐benefit analysis during their review of early phase trials and that they can only approve trials when those reviews determine that there is an appropriate balance between potential risks and benefits. They can only responsibly determine this when they carefully vet the quality of the evidence used to support the inferences that sponsors and investigators draw from the evidence.

The results presented above are subject to several limitations. First, we recognize the potential for social desirability bias in which respondents over‐report socially desirable responses such as doing a good job conducting risk‐benefit analyses or underreport undesirable responses such as not being prepared to conduct risk‐benefit analysis. Second, the results presented above may not be generalizable to IRBs outside of either medical schools or research‐intensive hospitals (e.g., to commercial IRBs). Third, the perceptions and beliefs of IRB chairs may not represent the views and opinions of IRB members themselves. Fourth, as discussed above, our respondents appear to slightly overrepresent the lowest tier of institutions in terms of NIH funding, those in hospitals and those located in the South. Fifth, we did not ask how long respondents were at their institution and thus were unable to assess whether an IRB chair's tenure at their institution affected their responses.

In conclusion, the majority of IRB chairs report that risk‐benefit analysis is conducted well despite frequently reporting they are not very well prepared to do so. Contributory factors to a lack of preparedness potentially include information barriers, low volumes of proposals to review and other challenges. IRB chairs feel additional measures would be helpful in conducting risk‐benefit analysis for early phase clinical trials. Future research evaluating potential tools and expanding the study pool to include commercial IRBs is warranted.

## ACKNOWLEDGMENT

The authors would like to acknowledge financial support for this research through a grant from the National Institutes of Neurologic Diseases and Stroke, NINDS 5 R01 NS119622‐3.

## CONFLICT OF INTEREST STATEMENT

Spencer Phillips Hey is the company founder and a shareholder of Prism Analytic Technologies.

## Supporting information

The appendix is available in the “Supporting Information” section for the online version of this article and via *Ethics & Human Research*'s “Supporting Information” page: https://www.thehastingscenter.org/supporting-information-ehr/.

Supporting information
