# Supplementary material for: Challenges of Conducting Risk‐Benefit Analysis of Early Phase Clinical Trials: Results of a National Survey of IRB Chairs
Source: Ethics Hum Res. 2025 Nov 12;47(6):2–12. doi: 10.1002/eahr.60024 (PMC12609048; doi:10.1002/eahr.60024)
Supplement: Supplementary file 1 — Supporting information [file EAHR-47-2-s001.pdf]

National Survey of IRB Chairs Regarding the Review of Risks and Benefits of Early Phase Clinical Trials

2022

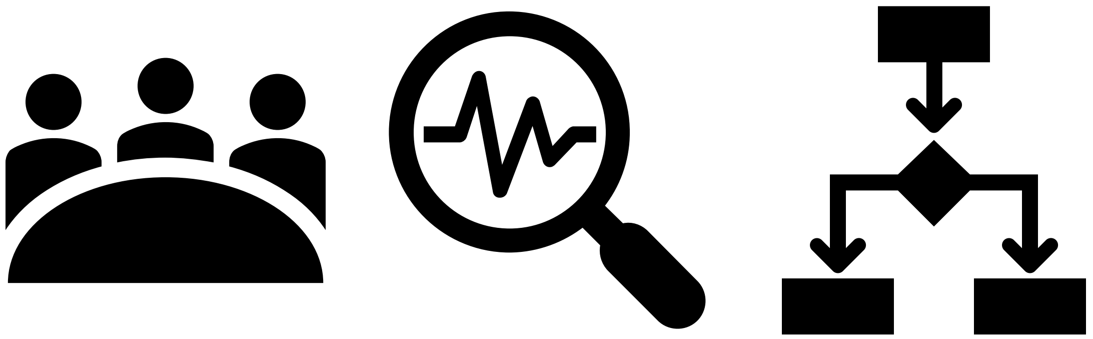

Research Team:
University of California Davis
McGill University
University of Colorado School of Medicine
Case Western Reserve University School of Medicine
Harvard Medical School

Conducted by:
Center for Survey Research
University of Massachusetts-Boston

SURVEY INSTRUCTIONS

- Your responses are completely confidential!
- Your participation in this study is voluntary.
- If there is a question you would rather not answer, feel free to skip it and go on to the next question.
- Please return your completed survey in the enclosed postage-paid envelope to the Center for Survey Research. If you have any questions about this survey or do not wish to participate, please call Dragana Bolcic-Jankovic at the Center for Survey Research at 1-800-492-5845.

Completion and return of this survey confirms your consent to participate.

PLEASE COMPLETE THIS SECTION FIRST

S1. Before you begin, have you been a chair or a co-chair/vice-chair of an IRB in the past two years?

☐ 1 Yes, Chair
☐ 2 Yes, Co-Chair/Vice-Chair
☐ 3 No → **IF NO, DO NOT CONTINUE.** Please return the questionnaire in the envelope provided and we will remove your name from our list. This will ensure that you are not re-contacted to participate in the survey. Thank you!

S2. Do you have any experience reviewing early phase clinical trials?

☐ 1 Yes → IF YES, PLEASE SEE KEY DEFINITIONS THEN COMPLETE SURVEY
☐ 2 No → **IF NO, DO NOT CONTINUE.** Please return the questionnaire in the envelope provided and we will remove your name from our list. This will ensure that you are not re-contacted to participate in the survey. Thank you!

KEY DEFINITIONS

- In this survey we ask about "**your IRB**." Please think about the IRB committee/panel that you chair/co-chair/vice-chair.
- **Early Phase Trials** are usually Phase 1 but sometimes Phase 2 clinical trials of drugs or biologics being studied for the first time in patients with a particular condition.
- **Preclinical efficacy studies** are laboratory studies, often in model organisms, aimed at establishing that a new drug has the potential to effectively treat a human illness.
- **Risk-Benefit Analysis** for the purposes of this survey is meant to refer to the process used by your IRB to determine whether or not the ratio of risks and benefits for a given clinical trial is reasonable. By reasonable, we mean that in federal regulations IRBs are required to determine that the research has a "reasonable ratio" between the risks and benefits of conducting a study.
- **An investigational new drug (IND)** application or request must be filed with the FDA when researchers want to study a drug in humans.
- **Neurological diseases** are diseases or disorders that are regularly treated by a neurologist and include but are not limited to Epilepsy and Seizures, Stroke, Amyotrophic Lateral Sclerosis (ALS), Alzheimer's Disease and Dementia and Parkinson's Disease.

Section A. IRB Characteristics

A1. Which of the following best describes the setting of your most recent IRB service?

(Select only one)

☐ 1 Medical school/Academic medical center IRB

☐ 2 Hospital Based IRB affiliated with a medical school/academic health center

☐ 3 Hospital Based IRB not affiliated with a medical school/academic health center

☐ 4 Commercial/Third party IRB

☐ 5 Other IRB (please specify) _______________

A2. In your most recent year as chair or co-chair/vice-chair of an IRB, about how many new protocols of any kind received a full review from your IRB? *(**Reminder:** When answering about **"your IRB,"** please think just about the IRB committee/panel that you chair/co-chair/vice-chair.)*

☐ 1 Less than 100

☐ 2 100-199

☐ 3 200-499

☐ 4 500 or more

B. Early Phase Clinical Trials

***Reminder: Early Phase Clinical trials** are usually Phase 1 but sometimes Phase 2 clinical trials of drugs or biologics being studied for the first time in patients with a particular condition.*

B1. In your most recent year of IRB service as chair or co-chair/vice-chair, about how many new protocols for early phase clinical trials came before your IRB?

☐ 0 None

☐ 1 1-4

☐ 2 5-9

☐ 3 10 or more

B2. Compared to later phase trials, how difficult is it for your IRB to conduct a risk-benefit analysis of early phase clinical trials?

☐ 1 A lot more difficult

☐ 2 A little more difficult

☐ 3 About the same

☐ 4 A little easier

☐ 5 A lot easier

B3. When conducting a risk-benefit analysis of early phase clinical trials, to what extent does your IRB rely on…?

	To a great extent	To some extent	To a very little extent	Not at all
B3a. Information from relevant pre-clinical studies that is included in the Investigator's Brochure provided by sponsor	☐ 1	☐ 2	☐ 3	☐ 4
B3b. Information from additional pre-clinical studies that supplements the information in the Investigator's Brochure provided by sponsor	☐ 1	☐ 2	☐ 3	☐ 4
B3c. Expertise of IRB members	☐ 1	☐ 2	☐ 3	☐ 4
B3d. Ad hoc expertise from outside your IRB	☐ 1	☐ 2	☐ 3	☐ 4

B4. How important are each of the following when conducting risk-benefit analysis of early phase clinical trials? *(Check one for each)*

	Extremely important	Very important	Somewhat important	Not very important	Not at all important
B4a. Having a DSMB overseeing the study	☐ 1	☐ 2	☐ 3	☐ 4	☐ 5
B4b. Having an IND from the FDA	☐ 1	☐ 2	☐ 3	☐ 4	☐ 5
B4c. Knowing that the study was funded by industry, e.g., a drug or biotech company	☐ 1	☐ 2	☐ 3	☐ 4	☐ 5
B4d. Knowing that the study was funded by a non-industry source, e.g., NIH, Foundation	☐ 1	☐ 2	☐ 3	☐ 4	☐ 5

B5. To what extent does the fact that the FDA has granted an IND for an early phase clinical trial give your IRB assurance that the risks and benefits are favorably balanced?

☐ 1 To a great extent
☐ 2 To some extent
☐ 3 To a very little extent
☐ 4 Not at all

B6. Overall, how good of a job does your IRB do when conducting a risk-benefit analysis of early phase clinical trials?

☐ 1 Excellent
☐ 2 Very Good
☐ 3 Good
☐ 4 Fair
☐ 5 Poor

B7. When conducting a risk-benefit analysis of <u>early phase clinical trials</u>, which of the following is most often the case for your IRB? *(Check one)*

☐ 1 The IRB prioritizes protecting patients from the risks posed by the research
☐ 2 The IRB prioritizes ensuring that patients have access to investigational drugs/biologics
☐ 3 The IRB prioritizes advancing the development of new drugs/ biologics

B8. Overall, how prepared is your IRB to do each of the following for protocols for <u>early phase clinical trials</u>? By prepared we mean your IRB has sufficient expertise and information to…

	**Very prepared**	**Mostly prepared**	**A little prepared**	**Not at all prepared**
B8a. Analyze the potential direct benefits for participants	☐ 1	☐ 2	☐ 3	☐ 4
B8b. Analyze the potential knowledge benefits of the trial	☐ 1	☐ 2	☐ 3	☐ 4
B8c. Analyze the risks	☐ 1	☐ 2	☐ 3	☐ 4
B8d. Determine if the risks have been adequately minimized	☐ 1	☐ 2	☐ 3	☐ 4
B8e. Determine that the risks are reasonable in relation to the benefits	☐ 1	☐ 2	☐ 3	☐ 4

B9. When your IRB is conducting a risk-benefit analysis for <u>early phase clinical trials</u>, how often is it the case that…?

	**Never**	**Rarely**	**Sometimes**	**Usually**	**Always**
B9a. Your IRB is satisfied with the summary of pre-clinical studies' results found in the Investigator's Brochure and study protocol	☐ 1	☐ 2	☐ 3	☐ 4	☐ 5
B9b. Pre-clinical studies provide sufficient information to analyze the potential benefits	☐ 1	☐ 2	☐ 3	☐ 4	☐ 5
B9c. Pre-clinical studies provide sufficient information to analyze the risks	☐ 1	☐ 2	☐ 3	☐ 4	☐ 5
B9d. The potential benefits are often much easier to analyze than the risks	☐ 1	☐ 2	☐ 3	☐ 4	☐ 5
B9e. Members of your IRB voice concerns about the strength of the support reported in preclinical studies	☐ 1	☐ 2	☐ 3	☐ 4	☐ 5

C. Early Phase Clinical Trials for Neurological Diseases

Reminder: Neurological diseases *are diseases or disorders that are regularly treated by a neurologist and include but are not limited to Epilepsy and Seizures, Stroke, Amyotrophic Lateral Sclerosis (ALS), Alzheimer's Disease and Dementia and Parkinson's Disease.*

C1. Does your IRB review protocols for neurological clinical trials?

☐ 1 Yes
☐ 2 No ➡ **IF NO SKIP TO SECTION D**

C2. Do any current members of your IRB have specific scientific and/or clinical expertise in neurological diseases?

☐ 1 Yes
☐ 2 No

C3. When your IRB reviews studies pertaining to neurological diseases, how often does your IRB seek ad hoc expertise in neurological diseases?

☐ 1 Never
☐ 2 Rarely
☐ 3 Usually
☐ 4 Always

C4. In your most recent year of IRB service as chair or co-chair/vice-chair, about how many new protocols for <u>early phase neurological clinical trials</u> came before your IRB?

☐ 0 None ➡ **IF NONE SKIP TO QUESTION C8**
☐ 1 1-4
☐ 2 5-9
☐ 3 10 or more

C5. In the process of risk-benefit analysis for <u>early phase neurological clinical trials</u>, does your IRB discuss the pre-clinical studies that support the clinical trial to be considered for approval?

☐ 1 Yes
☐ 2 No ➡ **IF NO SKIP TO QUESTION C7**

C6. When conducting a risk-benefit analysis of <u>early phase neurological clinical trials</u>, to what extent does your IRB assess whether…?

	To a great extent	To some extent	To a very little extent	Not at all
C6a. The preclinical studies may have produced exaggerated effect sizes and/or false positive findings	☐ 1	☐ 2	☐ 3	☐ 4
C6b. The key preclinical studies have been successfully replicated	☐ 1	☐ 2	☐ 3	☐ 4
C6c. The preclinical studies accurately simulate patients and the conditions under which they will receive the drug (appropriate animal model)	☐ 1	☐ 2	☐ 3	☐ 4
C6d. The drug targets an important mechanism in the neurological disease	☐ 1	☐ 2	☐ 3	☐ 4

C7. Besides standard templates used to review all studies, does your IRB use an additional standardized/structured process, such as a multi-item checklist, when conducting a risk-benefit analysis for <u>early phase neurological clinical trials</u>?

☐ 1 Yes
☐ 2 No

C8. How much responsibility do each of the following have in determining whether there is a reasonable ratio between the risks and potential benefits of an <u>early phase neurological clinical trial</u>?

	A lot of responsibility	Some responsibility	Very little responsibility	No responsibility
C8a. The IRB members who are <u>assigned</u> to the protocol	☐ 1	☐ 2	☐ 3	☐ 4
C8b. The IRB members who are <u>not assigned</u> to the protocol	☐ 1	☐ 2	☐ 3	☐ 4
C8c. The IRB Chair	☐ 1	☐ 2	☐ 3	☐ 4
C8d. The FDA	☐ 1	☐ 2	☐ 3	☐ 4

C9. How helpful would each of the following be to your IRB when reviewing early phase neurological clinical trials?

	Not at all helpful	A little helpful	Mostly helpful	Very helpful	*Not applicable*
C9a. Having a standardized process for conducting risk-benefit analyses	☐ 1	☐ 2	☐ 3	☐ 4	☐ 7
C9b. Having a computer program that summarizes the published literature on the benefits and potential risks of the drugs or biologics being studied	☐ 1	☐ 2	☐ 3	☐ 4	☐ 7
C9c. Having specific guidance from the federal Office of Human Research Protections	☐ 1	☐ 2	☐ 3	☐ 4	☐ 7
C9d. Having an outside group such as a scientific review committee conduct a risk-benefit analysis and advise the IRB	☐ 1	☐ 2	☐ 3	☐ 4	☐ 7
C9e. Having additional training for IRB members regarding the review of early phase neurological clinical trials	☐ 1	☐ 2	☐ 3	☐ 4	☐ 7

Section D. Demographics

D1. What is your gender? Do you identify as…? *(Select one)*

☐ 1 A woman
☐ 2 A man
☐ 3 Other, please specify: ______________

D2. Please indicate your race/ethnicity: *(Check all that apply)*

☐ 1 American Indian or Alaskan Native
☐ 2 Asian/Pacific Islander
☐ 3 Black
☐ 4 Hispanic
☐ 5 White
☐ 6 Other, please specify: ______________

D3. Have your ever been a Principal Investigator or Co-Investigator on an early phase clinical trial in any disease area?

☐ 1 Yes
☐ 2 No

Thank you for taking the time to complete this important survey.

In the space below please provide any comments or insights regarding IRB reviews about early phase clinical trials for neurological diseases that you feel is important for us to know.

Please comment here.

**RETURN INSTRUCTIONS**
Please return your completed questionnaire in the postage-paid envelope provided. If you misplaced the envelope, please send your questionnaire to: **Center for Survey Research** 100 Morrissey Boulevard, Boston, MA 02125.
